# IgG4-related diseases involving pleura: a case report and literature review

**DOI:** 10.3389/fmed.2023.1247884

**Published:** 2023-11-27

**Authors:** Anli Zuo, Xinyi Liu, Zihan Guo, Yunxiu Jiang, Degan Lu

**Affiliations:** Department of Respiratory, The First Affiliated Hospital of Shandong First Medical University and Shandong Provincial Qianfoshan Hospital, Shandong Institute of Respiratory Diseases, Shandong Institute of Anesthesia and Respiratory Critical Medicine, Jinan, China

**Keywords:** IgG4-related disease, idiopathic pleural lesions, pleural effusion, diagnosis, treatment, case report

## Abstract

Immunoglobulin G4-related disease (IgG4-RD) is a systemic fibro-inflammatory disease with the potential to involve virtually all organs, including the pancreas, kidneys, lungs, and pleura, amongst others. IgG4-RD pleural involvement may cause diverse complications such as pleural effusion, pleural thickening, pleural nodules, and additional lesions, which can be presented in many clinical diseases. However, isolated cases of pleurisy are still rare in IgG4-RD. We report a 72-year-old patient who was admitted to our hospital with cough, expectoration, and fatigue. He had a right-sided pleural effusion, and the tissue evaluation of the pleural biopsy by medical thoracoscopy met the diagnostic criteria of IgG4-RD. His serum IgG4 levels were elevated and he was finally diagnosed with IgG4-RD pleural involvement. He was subsequently started on prednisone 40 mg daily and his pleural effusion was almost disappeared 2 weeks later. This paper reported a case of IgG4-RD who had exclusive involvement of the pleura and highlighted the significance of considering IgG4-RD as a potential diagnosis in patients with unexplained pleural effusion.

## 1 Introduction

Immunoglobulin G4-related disease (IgG4-RD) is a systemic fibro-inflammatory disease characterized by lymphoplasmacytic infiltration of IgG4-positive cells in tissues, with or without serum IgG4 elevation (1). Initially, the descriptions of IgG4-RD primarily focused on its autoimmune pancreatitis. However, it has now been recognized as a chronic and multisystemic disorder. IgG4-RD can affect either a single or multiple organs and systems, including but not limited to salivary glands, lacrimal glands, thyroid gland, liver, kidney, and lung. When IgG4-RD affects these organs, it often leads to enlargement or swelling of the involved tissues (2). In severe cases, IgG4-RD may cause dysfunction in vital organs (3). IgG4-RD has the potential to involve all serous membranes throughout the body, but pleural involvement of IgG4-RD is relatively uncommon. When the pleura is affected in IgG4-RD, it can present with various manifestations, including pleural thickening, pleural nodules, and pleural effusion (2). A prospective cohort study found that pleural involvement occurred in 18 out of 248 (7.26%) patients with IgG4-RD. Among these cases, only 4 (1.61%) patients presented with pleural effusion. These findings suggest that pleural effusion remains a relatively rare finding in individuals with IgG4-RD (4). Therefore, pleural effusion remains an uncommon finding in IgG4-RD patients.

Herein, we present a case report of IgG4-RD accompanied by the presence of right pleural effusion, highlighting the necessity of including IgG4-RD in the etiological diagnosis of idiopathic pleural effusion.

## 2 Case presentation

**Figure 1:**
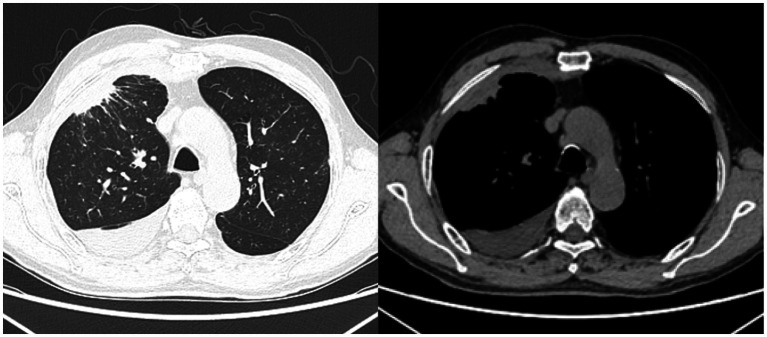
Chest CT on admission: there are patchy lesions observed in the upper lobe of the right lung, in conjunction with a right-sided pleural effusion accompanied by pleural thickening.

**Figure 2:**
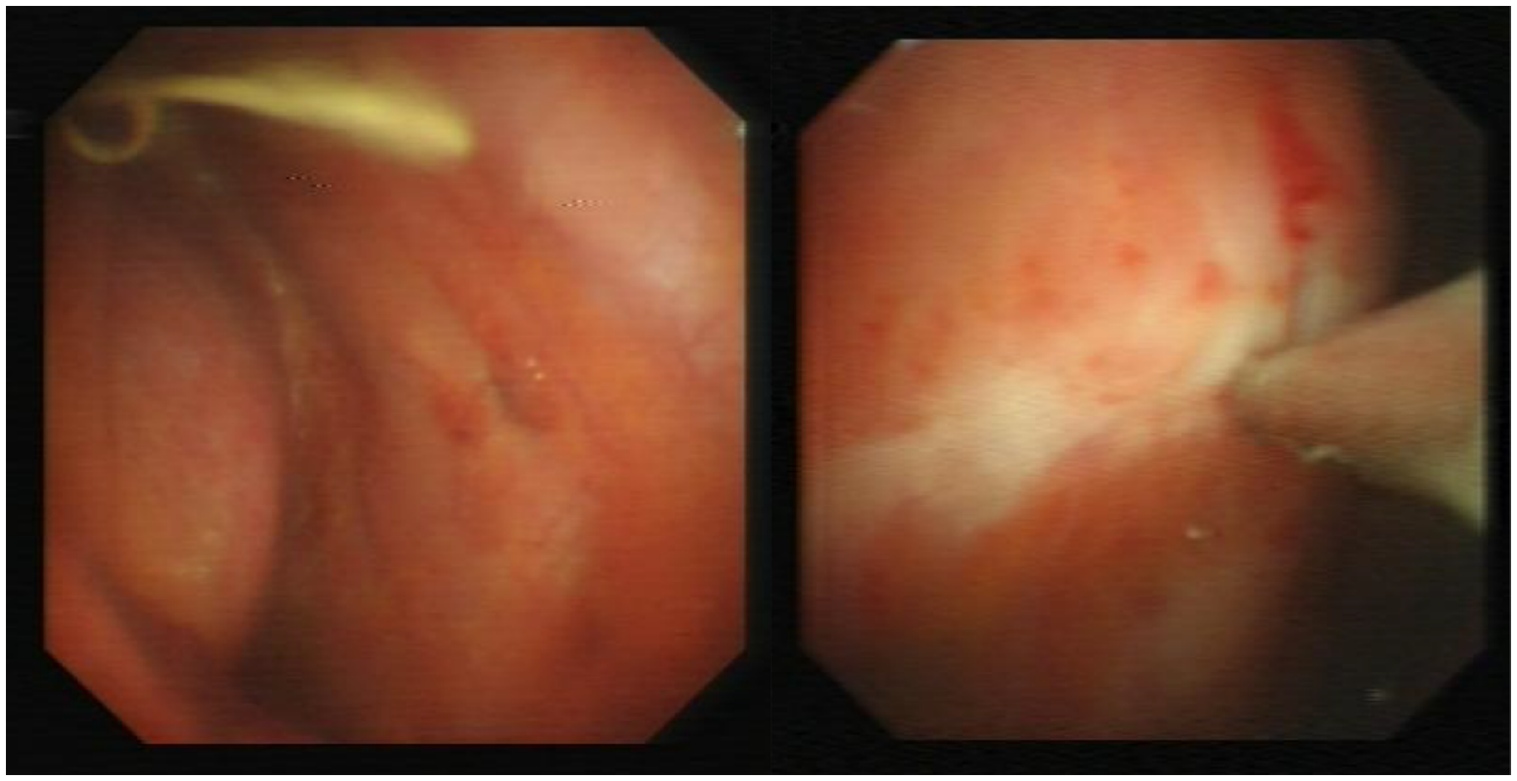
Thoracoscopic findings: parietal pleural hyperemia and white fibrotic plaque.

**Figure 3:**
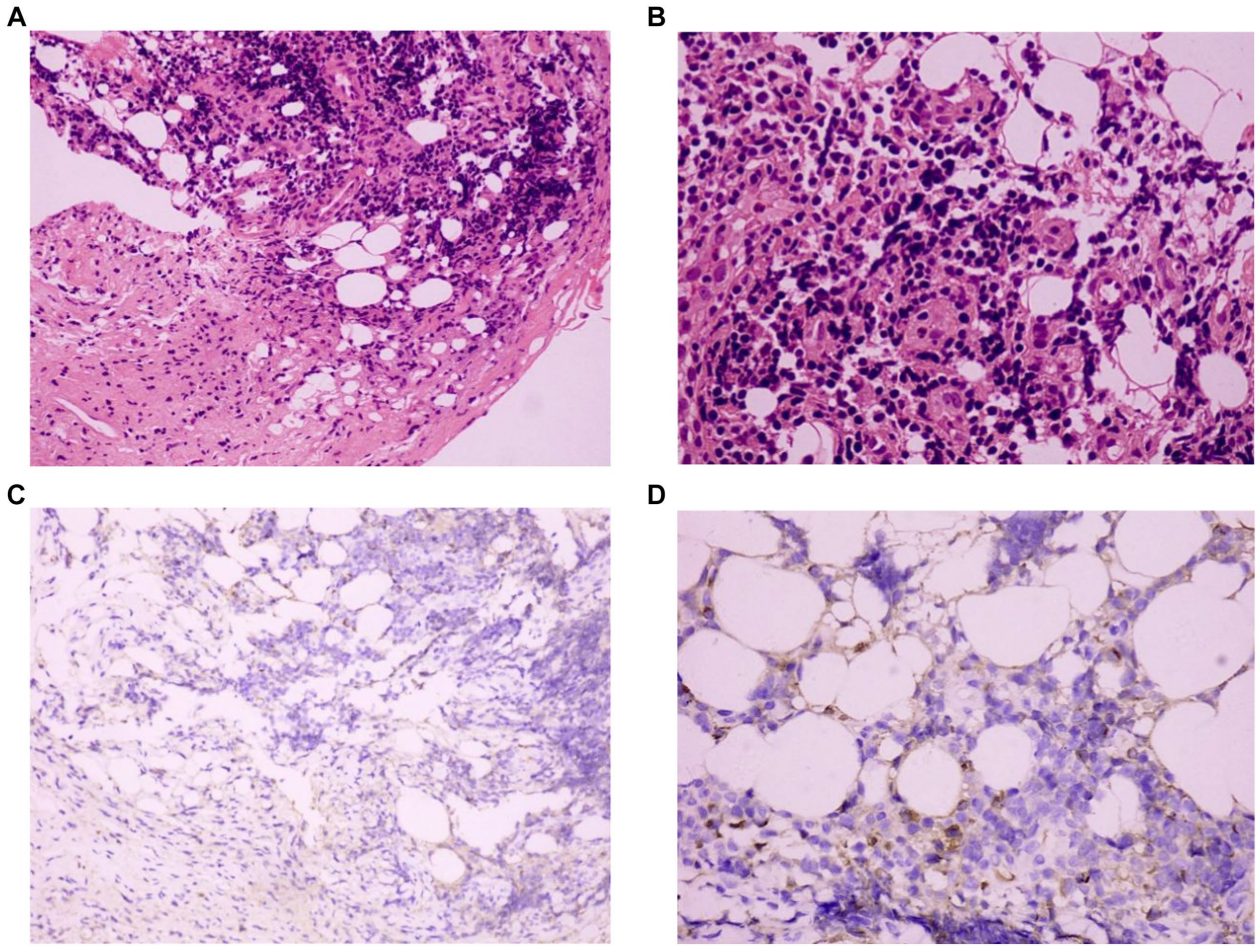
Histopathological evaluation of the pleura: **(A)** Hematoxylin and eosin staining (HE×200), **(B)** HE (×400): the right parietal pleural biopsy specimen reveals the presence of lymphoplasmacytic and plasma cell infiltration. **(C)** IgG4 immunohistochemical staining (×200), **(D)** IgG4 immunohistochemical staining (×400): the number of IgG4-positive plasma cells was 20/high-power field (HPF), and an IgG4/IgG ratio of 40–50%.

A 72-year-old Chinese man was admitted to our hospital with symptoms of fatigue, cough, and expectoration for half a month. He had a history of well-controlled bronchial asthma but no history of alcoholism, asbestos exposure, autoimmune disease or tuberculosis (TB). He was currently smoking 10 cigarettes per day. Upon admission, his body temperature, pulse rate, respiratory rate and blood pressure were 36.5°C, 80/min, 20/min, and 120/85 mmHg, respectively. Bronchoscopy and bronchoalveolar lavage outside the hospital showed no microorganisms, and cytological analysis ruled out malignancy. Laboratory tests on admission showed: white blood cell count 7.97 × 10^9^/L (neutrophils accounted for 74.1%), hemoglobin 110 g/L (130–175 g/L), platelet 358 × 10^9^/L (125–350 × 10^9^/L), C-reactive protein (CRP) 60 mg/L (0–3.48 mg/L), albumin 32.30 g/L (35–55 g/L), and globulin 46.50 g/L (20–35 g/L). Serum tumor marker tests, rheumatic immune series, anti-neutrophil cytoplasmic antibody (ANCA) and interferon-γ release test (TSPOT.TB) were all normal. Further imaging studies, including echocardiography, abdominal ultrasound (US), US of thyroid glands, the parotid and bilateral submandibular glands, and abdomen computed tomography (CT) scan showed no abnormalities. A chest CT revealed patchy lesions in the upper lobe of the right lung, as well as a right pleural effusion with pleural thickening (Figure 1). Right thoracentesis was performed, and analysis of the pleural fluid showed a predominance of lymphocytes (80%). Bacterial culture, polymerase chain reaction (PCR) tests for *Mycobacterium tuberculosis*, and cytology analysis were all negative. Video-assisted thoracoscopic surgery was subsequently performed (Figure 2) and histological examination of pleural specimen showed lymphoplasmacytic infiltrates accompanied by fibrous tissue hyperplasia. Immunohistochemical staining revealed the presence of IgG4-positive plasma cells [20/high-power field (HPF)], with a high IgG4/IgG ratio of 40–50% (Figure 3). Serum levels of immunoglobulin E (Ig E), IgA, IgM, IgG, and IgG4 were 235 IU/mL (<100 IU/mL), 2.59 g/L (0.7–4.0 g/L), 1.02 g/L (0.4–2.3 g/L), 26 g/L (7–16 g/L), and 5.88 g/L (0.03–2.01 g/L), respectively.

**Figure 4:**
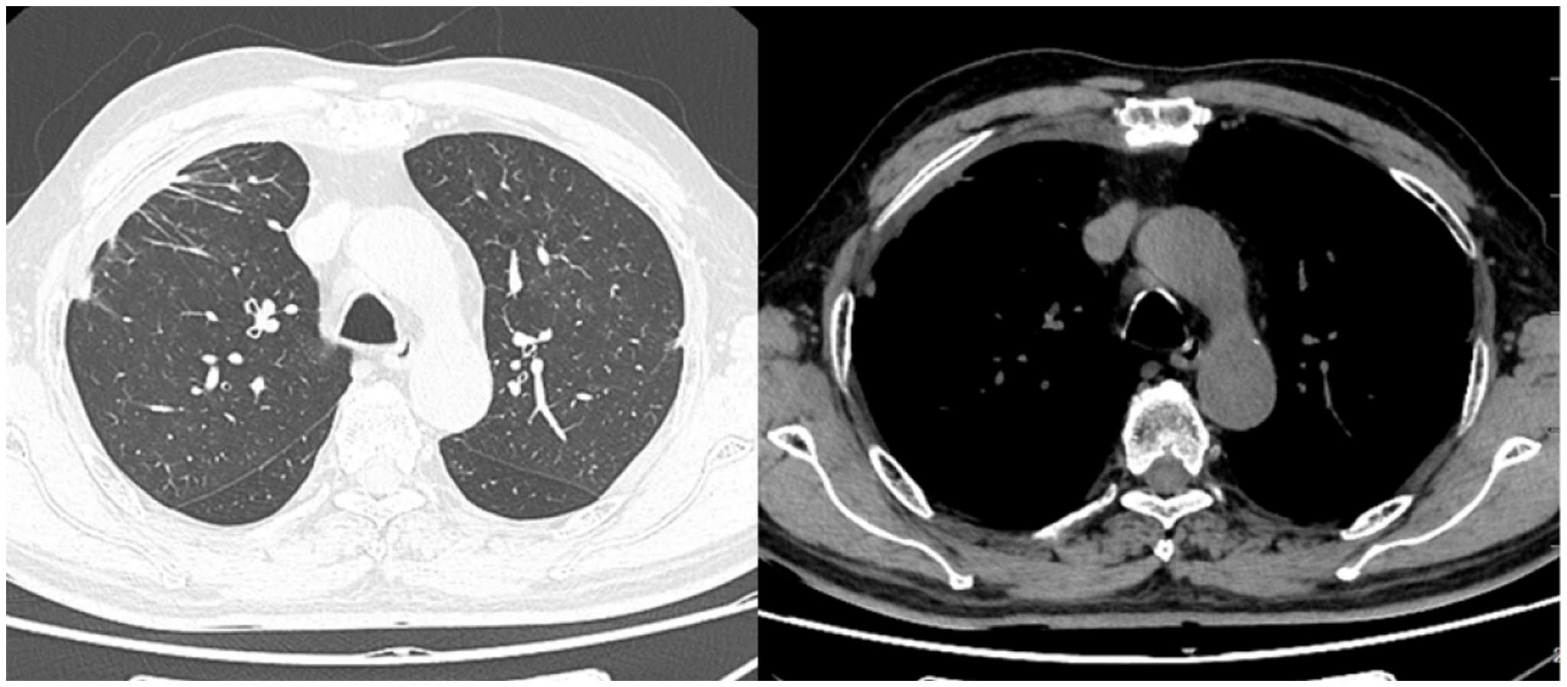
Chest CT after treatment: patchy lesions and pleural effusion in the right lung almost disappeared.

Based on these findings, a diagnosis of pleural involvement of IgG4-RD was made. The patient was commenced on prednisone 40 mg daily and a follow-up CT 2 weeks later showed significant improvement with almost complete resolution of the lung lesions and pleural effusion (Figure 4). Serum IgG4 level decreased to 3.09 g/L and CRP decreased to within the normal range. The patient was discharged with continued oral prednisone and was currently being followed up.

## 3 Discussion

IgG4-RD is indeed a fascinating clinical entity. It was first reported in Japan and has been found to be involved in a proportion of patients with undiagnosed pleural effusion (5). Kasashima et al. (6) described that 8 out of 22 (36.36%) patients with idiopathic pleural lesions satisfied the comprehensive pathological diagnostic criteria for IgG4-related pleural lesions (IgG4-PL). Additionally, Murata and colleagues were the first to report on the incidence of IgG4-RD in cases of idiopathic pleural effusion. They found that among 35 cases of pleural effusion with unidentified etiology, 12 (34.29%) were confirmed to be associated with IgG4-RD following pleural biopsy (7). The main pattern of pleural involvement in IgG4-RD includes pleural nodules or thickening, while pleural effusion is relatively rare (4). However, it is important for physicians from diverse specialties to maintain heightened awareness of IgG4-RD as a potential underlying cause when evaluating cases of pleural manifestations.

We conducted a search of MEDLINE database for English literature from the beginning year of inclusion to September 2023, using the keywords: “IgG4 related diseases” AND “pleural effusion” OR “pleural involvement.” We analyzed a total of 37 cases of IgG4-RD with pleural involvement after excluding studies with mismatched clinical data and non-case reports (Table 1). The average age of the collected cases was 65.14 ± 15.31 years old, and 29 (78.38%) of them were male. In all cases, exudative pleural effusion was presented. Of these, 19 cases (51.35%) had unilateral pleural effusion, with a predominance on the right side, as supported by our case report.

**Table 1:** Reported cases of IgG4-RD with pleural lesions.

Case No.	Gender	Age	Serum IgG4 (mg/dL)	Pathological findings	IgG4-positive plasma cells (/HPF)	IgG4/IgG	Cytology of pleural effusion	Pleural thickening	Pleural nodules	Pleural effusion	Extra-pulmonary organ involvement	Treatment	Treatment response
1 (8)	M	63	324	A large number of lymphoplasmacytic infiltrates	50	>40%	reactive mesothelial cells and lymphocytes	Right	–	Right exudate	N	PSL	Good response
2 (9)	M	75	1310	Lymphoplasmacytic infiltration	80	40% ~ 50%	Lymphocytes 83.5%	–	–	Left exudate	N	PSL	Good response
3 (2)	M	78	3404	Lymphoplasmacytic infiltration	10	40%	Lymphocytes, 79.2%; neutrophils, 12.9%	–	–	Right exudate	N	PSL	Good response
4 (10)	M	46	142	Lymphoplasmacytic infiltration	10	40%	A predominance of lymphocytes (68%)	–	Left	Bilateral exudate	N	PSL	Good response
5 (11)	M	75	1170	N/D	N/D	N/D	Lymphocytes %NA	–	–	Right exudate	Y	PSL	Good response
6 (11)	M	68	NA	Lymphoplasmacytic infiltration	N/D	N/D	Lymphocytes 96%	–	–	Bilateral exudate	N	PSL	Good response
7 (11)	M	84	1420	N/D	N/D	N/D	Lymphocytes 88%	–	–	Bilateral exudate	N	PSL + AZA	Good response
8 (11)	M	68	297	Lymphoplasmacytic infiltration	N/D	N/D	Lymphocytes 85%	–	–	Bilateral exudate	N	PSL + RTX	Good response
9 (12)	F	70	637	Lymphoplasmacytic infiltration	96	55.20%	Lymphocytes were dominant	–	–	Left exudate	Y	PSL	Good response
10 (13)	M	65	299	Lymphoplasmacytic infiltration	10	22.40%	Lymphocytes were dominant	Right	–	Right exudate	N	PSL	Good response
11 (14)	M	81	233	Dense lymphoplasmacytic infiltration with mild eosinophilic infiltration	>50	<40%	Numerous plasma cells	–	–	Bilateral exudate	N	PSL	Good response
12 (15)	M	43	248	Lymphoplasmacytic infiltration	0.24	N/D	Lymphocytes were dominant	–	–	Bilateral exudate	Y	PSL	Good response
13 (16)	M	66	264	Lymphoplasmacytic infiltration	N/D	50%	Lymphocytes 75%	–	–	Bilateral exudate	Y	PSL + RTX	Good response
14 (17)	M	64	275	Lymphoplasmacytic infiltrates with laminar fibrosis	>20	>50%	a predominance of mononuclear cells	–	–	Bilateral exudate	N	PSL	Good response
15 (18)	F	61	174	Lymphoplasmacytic infiltration	>50	>40%	The total cell number	Right	–	Right exudate	Y	PSL	Good response
							(1.763 × 10^12^/L)						
16 (19)	M	16	1650	N/D	N/D	N/D	No malignant cells	–	–	Bilateral exudate	Y	PSL + AZA	Partial response
17 (20)	M	56	398	Lymphoplasmacytic infiltrates with laminar fibrosis	N/D	>50%	45% lymphocytes, 11% neutrophils, 9% eosinophils and 34% monocytes	–	–	Right exudate	Y	PSL	Good response
18 (21)	F	70	270	Lymphoplasmacytic infiltration	50–60	>50%	Lymphocytes were dominant	–	–	Bilateral exudate	N	PSL	Good response
19 (22)	M	78	760	Lymphoplasmacytic infiltration	100	70%	N/D	–	–	N/A	Y	PSL	Good response
20 (23)	F	72	>1500	Lymphoplasmacytic infiltration	>50	>40%	Lymphocytes were dominant	Left	–	Left exudate	Y	N/A	Good response
21 (24)	M	78	483	Lymphoplasmacytic infiltration	17.6	85.40%	A predominance of mononuclear cells	Bilateral	–	Bilateral exudate	N	diuretics	Good response
22 (25)	F	69	724	Dense lymphoplasmacytic infiltration with mild eosinophilic infiltration	286	46%	Lymphocytes were dominant	–	–	Left exudate	N	PSL	Good response
23 (26)	M	65	253	Lymphoplasmacytic infiltrates	70	>30%	NA	Bilateral	–	Right exudate	N	PSL	Good response
				With laminar fibrosis									
24 (27)	F	43	125	Lymphoplasmacytic infiltration	80	>40%	Lymphocytes were dominant (80% ~ 90%)	–	Right	Right exudate	Y	PSL	Good response
25 (28)	M	70	224	Lymphoplasmacytic infiltrates with laminar fibrosis	>50	N/D	A predominance of mononuclear cells	–	–	Right exudate	N	PSL	Good response
26 (29)	F	74	740	Lymphoplasmacytic infiltration	91	91%	A predominance of mononuclear cells	Bilateral	–	Bilateral exudate	N	PSL	Good response
27 (30)	F	29	136	Lymphoplasmacytic infiltration	30	92%	Lymphocytes were dominant (93%)	Bilateral	–	Right exudate	Y	PSL	Good response
28 (31)	M	64	570	Lymphoplasmacytic infiltrates with fibrosis	20–30	40%	N/D	Right	–	Right exudate	N	N/D	Good response
29 (32)	M	74	>201	Lymphoplasmacytic infiltration	N/D	40%	N/D	–	–	Right exudate	N	PSL	Good response
30 (33)	M	39	136	Lymphoplasmacytic infiltrates and phlebitis obliterans with laminar fibrosis	40	>50%	A predominance of mononuclear cells	Left	–	Left exudate	N	PSL	Good response
31 (34)	M	63	415	Lymphoplasmacytic infiltration	10	>40%	N/D	Bilateral	–	Right exudate	Y	PSL	Good response
32 (35)	M	74	242	Lymphoplasmacytic infiltration	>10	>40%	Lymphocytes 78%	–	–	N/D	N/D	PSL	Good response
33 (35)	M	86	817	Lymphoplasmacytic infiltration	>10	>40%	Lymphocytes 82%	–	–	N/D	N/D	PSL	Good response
34 (35)	M	65	1416	Lymphoplasmacytic infiltration	>10	>40%	Lymphocytes 71%	–	–	N/D	N/D	PSL	Good response
35 (35)	M	59	NA	Lymphoplasmacytic infiltration	>10	>40%	Lymphocytes 68%	–	–	N/D	N/D	PSL	Good response
36 (36)	M	81	848	Dense lymphoplasmacytic infiltration with mild eosinophilic infiltration	50	>40%	63% eosinophils	Right	–	Right exudate	N	PSL	Good response
37 (37)	M	78	2100	Lymphoplasmacytic infiltration	>20	40% ~ 50%	N/D	Bilateral	–	Bilateral exudate	N/D	PSL	Good response

F, Female; M, Male; N/D, Not determined; NA, Normal; Y, Yes; N, No; PSL, Prednisone; AZA, azathioprine; RTX, rituximab.

It seems to be interesting that our patient had a history of asthma, which aligns with previous case reports on this matter (21, 23). Kasashima et al. (6) also reported that 5 of 8 cases of IgG4-PL had a history of allergic diseases, including bronchial asthma, chronic bronchitis, and eosinophilic rhinitis. While allergic symptoms are more commonly observed in IgG4-RD patients with head and neck involvement (38), patients with pleural involvement also tend to have a history of allergies. Hence, the possibility of IgG4-RD should be considered in patients with idiopathic pleurisy combined with a history of allergy.

Regarding serology, it is worth noting that 34 (91.89%) of the patients in our collected cases exhibited elevated serum IgG4 levels (Table 1). Increased serum IgG4 is considered one of the criteria for a definitive diagnosis of IgG4-RD (39). Yang and colleagues also reported that serum IgG4 level was associated with the number of affected organs (40). However, it is important to be aware that elevated serum IgG4 levels can also be present in a range of other conditions, including healthy individuals, malignant neoplasms, infectious diseases, and other inflammatory conditions (8). Therefore, relying solely on serum IgG4 levels is insufficient for diagnosing or ruling out IgG4-RD. IgG4 antibodies may serve as markers for immune-mediated processes as part of more complex inflammatory diseases (8).

In IgG4-PL patients, it is typically observed that CRP levels are within normal range. However, in the cases we analyzed, 10 individuals demonstrated a mild increase in CRP levels (8, 13, 18, 20, 21, 27, 28, 33, 36, 37). It is important to highlight the contrast between IgG4-PL and conditions such as ANCA associated vasculitis and multicentric Castleman disease (MCD) when considering the levels of inflammatory markers like CRP. In these conditions, there is typically a marked increase in CRP levels (3, 41). However, in IgG4-PL patients, the increase in CRP levels is mild or not as pronounced (6). Substantial elevations in CRP levels are often indicative of the onset of infectious or inflammatory diseases that are similar to IgG4-RD (3). In addition, patients with higher CRP levels in IgG4-PL cases tended to have lower serum IgG4 levels (42), which was consistent with the patient we reported and the cases we collected.

It is noteworthy that elevated levels of adenylate deaminase (ADA) in pleural fluids have been reported in some IgG4-PL cases in the studies we retrieved (9, 11–13), although it was within the normal range in our patient. ADA is commonly used as a sensitive indicator for the diagnosis of tuberculous pleurisy in clinical settings. It is important to distinguish between IgG4-PL and tuberculosis when ADA levels are elevated, especially considering that glucocorticoids (GC) – the first-line treatment for IgG4-RD – may potentially contribute to the dissemination of tuberculosis infection (25). In the definitive diagnosis of these conditions, pleural effusion culture and pleural biopsy play crucial roles. However, the success rate of tuberculous bacterium culture in pleural effusion is low, necessitating the use of pleural biopsy to detect granulomatous lesions with higher sensitivity and specificity (12).

Our case needs to be differentiated from tuberculous pleurisy, malignant pleural effusion, and MCD. Acute symptoms such as fever, chest pain, and dry cough are often associated with tuberculous pleurisy (43). A combination of PCR, solid and liquid culture, TSPOT.TB, and pleural biopsy is necessary to differentiate between IgG4-PL and tuberculous pleurisy (5). This patient does not show any signs of tuberculosis. Based on the serological results, analysis of pleural fluid, pleural biopsy, and treatment response, the presence of malignant pleural effusion can reliably be ruled out in this patient. As an uncommon systemic lymphoproliferative disorder, MCD can lead to damage in multiple organs, which may resemble IgG4-RD (44). The age of onset and levels of serum CRP and interleukin-6 often help in distinguishing MCD from other conditions (5). Distinguishing between IgG4-RD and inflammatory myofibroblastic tumor (IMT) is also crucial. IMT is distinct from IgG4-RD due to its true neoplastic nature and the presence of fascicular proliferation of spindle cells. The number of IgG4-positive plasma cells is also helpful in making this distinction (45).

Although nonspecific, imaging serves as a crucial component of the diagnostic approach for various organs. In cases with pleural involvement, imaging manifestations may include pleural effusion, pleural thickening, pleural calcification, and pleural nodules (4). Radiological tools such as CT and US have high sensitivity for detecting diseased organs in IgG4-RD, especially when assessing multiorgan involvement. Among the cases we have collected, the extrapulmonary involvement was identified in 12 cases (32.43%) (Table 1), with imaging examinations playing an important role. Fluorodeoxyglucose-PET (FDG-PET) has been confirmed to be a reliable technique for evaluating IgG4-RD activity and detecting clinically asymptomatic IgG4-RD organ involvement in the thoracic region (lung or pleura) (46). However, exclusive reliance on FDG-PET to rule out pleural lesions is not advisable, as FDG accumulation may not always be present in the pleura of IgG4-RD patients with pleural involvement (20, 22, 23, 26). Therefore, it is important to be cautious when relying solely on FDG-PET findings to exclude pleural lesions. Occasionally, imaging findings can be “confusing.” For example, a case report by Ishikawa et al. (47) described a case of IgG4-RD mimicking lung cancer with pleural dissemination. The chest CT scan showed multiple nodules on the adjacent pleural surface, which were ultimately confirmed as IgG4-RD involving both lung and pleura. This highlights the need to differentiate between IgG4-PL and pleural metastasis in the presence of pulmonary nodules. In reality, lung cancer may co-exist with IgG4-RD pleural involvement (47), and IgG4-RD itself increases overall risk of developing cancer (48). Therefore, it is crucial to maintain vigilance for malignant tumors in patients with IgG4-RD.

Pleural lesions in IgG4-RD can sometimes be directly visualized via thoracoscopy. However, not all cases of IgG4-RD involving the pleura appear abnormal on thoracoscopic examination (12). Histopathological evaluation plays a crucial role in the diagnosis of IgG4-RD. Key histopathologic features include dense lymphoplasmacytic infiltration, obliterans phlebitis, laminar fibrosis, and mild to moderate tissue eosinophil infiltration (20). The presence of epithelioid cell granuloma, marked neutrophil infiltration, abscess, and necrosis may raise suspicion of alternative disorders (8). Pathological diagnosis facilitates clear differentiation of IgG4-RD from conditions such as sarcoidosis, connective tissue disease, malignant tumors, and others. In the cases we reviewed, almost all exhibited lymphoplasmacytic infiltration, only 4 cases (10.81%) had laminar fibrosis, and 3 cases (8.10%) had mild eosinophil infiltration (Table 1). While laminar fibrosis, obliterative phlebitis, and eosinophilic infiltration are uncommon in IgG4-RD lung involvement (49), both obliterated phlebitis and obliterated arteritis are more common in solid nodules and pleural lesions compared to other subtypes, potentially due to more pronounced diffuse sclerosing inflammation in these subtypes (45).

GC are the first-line therapeutic drugs for IgG4-RD (5). The dose of GC should be adjusted according to body weight and the extent of disease involvement. Prednisone at 30–40 mg/day is commonly effective in most cases and can be gradually tapered after 2–4 weeks if the disease is controlled. However, the response to hormonal therapy may vary between patients with pleural and non-pleural involvement, as pleural fibrosis can affect the effectiveness of hormones and lead to poorer prognosis with conventional dosing (6). In the cases analyzed (Table 1), corticosteroids were used as initial treatment, resulting in symptom improvement in most patients. Immunosuppressants or rituximab (RTX), a monoclonal antibody targeting CD20, may be considered in cases of recurrence, hormonal- related adverse reactions, or multiorgan involvement (11, 16). Surgical treatment may be effective for IgG4-PL patients who experience recurrent pleural effusion after GC combined with immunosuppressant therapy. Isolated IgG4-RD pleural involvement usually follows a benign course, and patients with minimal asymptomatic effusion can be monitored through radiographic follow-up (5).

## 4 Conclusion

In summary, IgG4-RD involving the pleura is a clinically uncommon and heterogeneous entity. The main patterns of pleural involvement consist of pleural nodules, pleural thickening, and pleural effusion. Medical thoracoscopy serves an important role in diagnosing pleural involvement in IgG4-RD, especially in cases of pleural effusion. A definitive diagnosis of IgG4-related pleural lesions relies on the histological identification of a lymphoplasmacytic infiltrate with abundant IgG4+ plasma cells, storiform fibrosis and obliterative phlebitis in the pleurae and on the exclusion of all other possible causes. The possibility of IgG4-RD should be considered in patients with unexplained pleural effusion charactered by the presence of IgG4-positive plasma cell in pleural biopsies. Generally, IgG4-PL respond well to systemic corticosteroid therapy.

## Data availability statement

The original contributions presented in the study are included in the article/supplementary material, further inquiries can be directed to the corresponding author.

## Ethics statement

Ethical review and approval was not required for the study on human participants in accordance with the local legislation and institutional requirements. Written informed consent from the patients was not required to participate in this study in accordance with the national legislation and the institutional requirements. Written informed consent was obtained from the individual(s) for the publication of any potentially identifiable images or data included in this article.

## Author contributions

AZ, XL, ZG, YJ, and DL provided the ideas for this case report. AZ and DL prepared and revised the manuscript. DL reviewed the article. XL, ZG, and YJ confirmed the authenticity of all original data. All authors have read and approved the final version of the manuscript.
